# Unveiling the potential of SLURP1 protein as a biomarker for prostate cancer screening

**DOI:** 10.3389/fonc.2024.1365615

**Published:** 2024-04-15

**Authors:** Tianyin Yang, Tianci Liu, Ting Lei, Tao Li, Na Liu, Man Zhang

**Affiliations:** ^1^ Clinical Laboratory Medicine, Beijing Shijitan Hospital, Capital Medical University, Beijing, China; ^2^ Beijing Key Laboratory of Urinary Cellular Molecular Diagnostics, Beijing, China; ^3^ Institute of Regenerative Medicine and Laboratory Technology Innovation, Qingdao University, Qingdao, China

**Keywords:** prostate cancer, urine, proteomics, SLURP1, biomarkers

## Abstract

**Background:**

Prostate cancer (PCa) develops slowly and lacks obvious symptoms in the early stage, which makes early screening and diagnosis difficult. Urine collection is simple and is an ideal source of biomarkers. In this study, we performed urinary proteomic studies in PCa patients to screen proteins and apply them to the non-invasive early diagnosis of PCa.

**Method:**

Urine samples from PCa patients, benign prostatic hyperplasia (BPH) patients and normal control group were collected. Mass spectrometry was used for proteomic analysis and screening target proteins. Western blot and enzyme-linked immunosorbent assay (ELISA) were used to verify the results. Correlations with clinical indicators were explored, and receiver operating characteristic (ROC) curves were drawn to evaluate the value of target proteins in PCa.

**Result:**

A total of 1065 proteins were identified. Urinary SLURP1 protein was significantly elevated in patients with PCa compared with normal controls and patients with BPH patients. Western blot and ELISA further verified the expression changes of SLURP1. The immunohistochemical staining results revealed a substantial increase in positive SLURP1 expression within PCa tumor tissue. Correlation analysis showed a positive correlation between the expression level of urine SLURP1 protein and serum PSA. ROC curve analysis of the SLURP1 protein in the urine of both normal individuals and PCa patients is determined to be 0.853 (95% CI=0.754 to 0.954).

**Conclusion:**

The concentration of SLURP1 protein in urine of PCa patients is increased, which can serve as a biomarker for screening PCa.

## Introduction

Prostate cancer (PCa) is one of the most common and deadly cancers that affect men, according to the World Cancer Report (1). In 2023, the American Cancer Society predicts that there will be over 1900000 new cases of malignant tumors and approximately 610000 deaths (2). PCa incidence and mortality have increased significantly in China due to aging populations, westernized diets, and other factors.Symptoms of PCa are not apparent at an early stage, and most patients are already in the middle to late stages when they are diagnosed (3). It is important to screen, diagnose, and treat cancer patients as early as possible in order to improve their survival rates (4, 5). As the final metabolite of blood, urine carries a variety of information about the occurrence, development, and prognosis of diseases of the urinary system (6). Urine has the advantage of being non-invasive and convenient to collect when compared to other bodily fluids, including serum.The prostate gland can release PCa cells and their metabolites into the urine (7–9). Consequently, by analyzing bioactive substances in urine, it is possible to undertake relevant research on the development and occurrence of PCa.

Secreted LY6/uPAR domain containing 1 (SLURP1) is a constituent of the lymphatic antigen-6 (Ly6) family. The genes belonging to the LY6 family are situated in the 8q24 chromosome region, a frequently observed site of amplification in human cancer, and are involved in various cellular processes such as proliferation, migration, cell-cell interactions, immune regulation, macrophage activation, and cytokine secretion (10, 11). The SLURP1 gene is situated on the q24.3 region of the long arm of chromosome 8 and exhibits expression in a diverse range of cells, with a particular emphasis on epithelial cells. SLURP1 has been identified as a regulator of epithelial cell growth, involvement in epithelial mesenchymal transition, programmed cell death, inflammation, and tumor development, and serves as a marker for late epithelial differentiation (12). Extensive research has demonstrated a significant correlation between the onset and progression of PCa and the impairment of prostate epithelial cells (13). The damage to prostate epithelial cells can be attributed to various physical, biochemical, and other factors, as well as the continuous infiltration of inflammatory cells. Consequently, this damage triggers subsequent repair and proliferation, ultimately leading to the development of intraepithelial neoplasia within the prostate (14). The occurrence of epithelial mesenchymal transition (EMT) in PCa has the potential to augment cellular invasiveness and metastatic capabilities, accelerate the pace and frequency of subcutaneous tumor development, and represents a crucial factor in the migration and invasion of cancerous cells (15). Currently, there is a lack of reported data regarding the expression of SLURP1 in benign prostatic hyperplasia (BPH) and PCa, leaving uncertainty regarding its viability as a diagnostic marker for PCa.

In this study, proteomic techniques were used to detect differential protein expression profiles in the urine of normal controls, BPH, and PCa. The investigation focused on the expression alterations of the SLURP1 protein, validating them in both urine and tissue samples. Additionally, the study analyzed the association between the SLURP1 protein and the initiation of PCa, thereby establishing a basis for future research in identifying specific biomarkers for the diagnosis of PCa.

## Materials and methods

### Patients

A total of 100 adult patients who underwent urology treatment at Beijing Shijitan Hospital between January 2022 and May 2023 were selected as the participants for this study. Among them, 50 patients were diagnosed with PCa. The inclusion criteria for this study were as follows: (1) PCa diagnosis confirmed through histopathological examination; (2) First-time diagnosis for both PCa and urology treatment; (3) All patients underwent radical prostatectomy. The exclusion criteria for this study were: (1) Patients with other malignant tumors; (2) Patients with comorbidities such as hypertension, diabetes, and coronary heart disease; (3) Patients who received preoperative radiotherapy and chemotherapy. A total of 50 individuals diagnosed with BPH were included in the study, with their diagnoses confirmed through prostate resection or puncture. Patients with comorbidities or pre-existing malignancies were excluded from the study, which focused on individuals presenting with BPH for the first time and undergoing urological treatment. Additionally, 32 healthy volunteers were selected from the physical examination center of our hospital during the same time frame. The healthy controls were carefully matched with the patients diagnosed with PCa and BPH in terms of age and gender.

The screening group comprised 23 patients diagnosed with PCa, 23 patients diagnosed with BPH, and 5 healthy controls. Similarly, the validation group consisted of 27 patients with PCa, 27 patients with BPH, and 27 healthy controls. Prior to conducting this research, the Ethics Committee of Beijing Shijitan Hospital thoroughly reviewed and approved the research plan. Furthermore, the collection of samples and clinical data was conducted with the informed consent of the research subjects, who willingly signed an informed consent form.

### Urine sample collection and processing

Healthy individuals, as well as patients with conditions like BPH and PCa, should collect and deliver their mid-morning urine sample to the laboratory within two hours. Urine samples are collected before urology treatment. Subsequently, the urine should be subjected to centrifugation at a rate of 500g/min for a duration of five minutes to eliminate cellular matter and fragments. The resulting supernatant should then be divided into equal portions, properly packaged, labeled, and subsequently frozen at a temperature of -80°C until required for further analysis.

### Identification and analysis of urine by mass spectrometry

Urine samples were obtained from the screening group for the purpose of conducting mass spectrometry analysis. To prepare the samples, 0.42g of urea powder was added to 1mL of urine, followed by shaking for a duration of 10 minutes. Subsequently, ultrafiltration was performed using a 10k ultrafiltration tube (Bio Rad, product number 161-0460, USA) at a centrifugal force of 12000g. The resulting filtrate was collected using a 15-minute inverted ultrafiltration tube, and the ultrafiltration tube was rinsed with an 8m UA solution before merging it with the collected samples. Finally, quantification of the samples was carried out using the Bradford method. The Thermo Orbitrap Fusion Lumis mass spectrometer was utilized for conducting mass spectrometry analysis in data acquisition mode (DDA), encompassing a comprehensive scan range of 300-1400m/z and a resolution of 120000FWHM. The resulting mass spectrometry raw files were subjected to processing using the MaxQuant software. Annotation analysis was retrieved through the UniProt database (www.uniprot.org). The study employed the following parameters: a precursor mass tolerance of 20 ppm, a fragment mass tolerance of 0.5 Da, a static modification of carbamidomethylation on cysteine residues, variable modifications including oxidation of methionine and acetylation of N-termini of peptides, allowance for a maximum of two missed cleavages, and a protein false discovery rate (FDR) was 1%. Median normalization was conducted on the original data to address experimental errors. The filtered data underwent imputation of missing values using the perseuse algorithm. Differentially expressed proteins were identified based on meeting the criteria of an average ratio-fold change > 1.5 and a p-value < 0.05 (16–18).

### Western blot

The differential target protein SLURP1 was analyzed via Western blotting. The initial step involved concentrating the urine sample using a 3K ultrafiltration tube (Millipore, Billerica, USA). We use the BCA protein quantification kit (Epizyme Biomedical,Shanghai, China) to measure the total protein concentration in enriched urine samples as kit manual. Each sample is standardized to a sample size of 30 micrograms, thereby maintaining uniformity in the total mass of each sample. Determine the sample volume for each sample by dividing the total sample mass by the sample concentration in order to achieve standardization of protein expression levels. Foldchange 15% SDS-PAGE separation gel was prepared for the purpose of conducting acrylamide gel electrophoresis. The transfer of the gel to a PVDF membrane was carried out using equipment provided by transblot apparatus (Bio-Rad, California, USA). The membrane was subsequently sealed using skim milk powder. To ensure proper sealing, the membrane was placed on a shaker for a duration of 2 hours. Following this, it was incubated overnight with a primary antibody (1:1000 dilution; Abcam, Cambridge, UK). To remove any excess antibody, the membrane was washed three times with TBST, with each wash lasting for 15 minutes. Subsequently, a second antibody (diluted by 1: 2000; Bios, Beijing, China), labeled with horseradish peroxidase, was added to the membrane and incubated at room temperature for a period of 2 hours. After the incubation, the membrane was washed three times with TBST. Finally, the detection of the desired components was achieved using enhanced chemiluminescence (ECL). The protein gel electrophoresis image was collected and analyzed by automatic chemiluminescence image analyzer (GenoSens 2000, QinXiang, Shanghai, China).

### Enzyme linked immunosorbent assay

In the validation group, 27 patients with PCa, 27 patients with BPH, and 27 healthy controls were included. We centrifuged the urine samples at 1000xg for 20 minutes at 2-8°C after they had been thawed. Supernatants from centrifuged samples were collected and immediately detected using a Human SLURP1 ELISA kit(Fine test, Wuhan, China). According to the kit instructions, the procedure was repeated three times per sample. By using a microplate reader(Thremo, Waltham, USA, we measured the absorbance at 450 nm (OD450). In order to calculate the target concentration of the sample, the OD value of the sample was substituted into the standard curve. Target protein expression units were ng/ml.

### Immunohistochemical staining

This validation group includes 3 BPH tissue samples as well as 3 PCa tissue samples. The sequential execution of the following steps is recommended in an academic context: paraffin embedding, slicing, grilling, and sectioning, followed by water treatment, antigen repair, blocking of endogenous peroxidase, serum blocking, application of 3% BSA onto the histochemical circle to ensure uniform coverage of the tissue, and finally sealing the sample at room temperature for a duration of 30 minutes. The initial antibody (Abcam, Cambridge, UK) should be prepared in the appropriate ratio and applied onto the slices. Subsequently, the slices should be incubated overnight at a temperature of 4°C. The glass slide ought to be immersed in pH 7.4 PBS and subjected to three rounds of shaking and washing in a decolorization shaker, with each round lasting five minutes. Following the slicing and drying process, HRP-labeled secondary antibodies(Bios, Beijing, China) should be added to fully cover the tissue and incubated at room temperature for a duration of 50 minutes. Finally, DAB staining should be performed, followed by restaining of the nucleus.

Following the process of dehydration and sealing, the tissue slices underwent scanning and analysis utilizing the Aipathwell digital pathological image analysis software (Developed by Wuhan Servicebio Technology Co., Ltd.). The evaluation criteria encompassed two aspects: (1) The positive cell ratio, calculated as the number of positive cells divided by the total number of cells, and (2) The positive cell density, determined by dividing the number of positive cells by the area of the tissue sample under examination.

### Statistics

Statistical analysis was performed using SPSS 25.0 and Graphpad Prism 9.0 software. The Student’s t-test was utilized to compare two groups that adhered to a normal distribution, while the Mann-Whitney U test was employed for comparing two groups that did not conform to a normal distribution. Additionally, a One-way ANOVA analysis was conducted to compare three groups. Pearson correlation analysis was employed to assess correlation. Receiver Operating Characteristic (ROC) curves were generated to evaluate the diagnostic efficacy of the target proteins. A significance level of *P*<0.05 was considered indicative of a statistically significant difference.

## Result

### Clinical characteristics

The clinical baseline data of pertinent detection indicators in patients diagnosed with BPH and PCa are compared, including the Gleason score from pathological biopsies in prostate cancer patients, as depicted in **Table 1**. The observed elevation in levels of total prostate specific antigen (tPSA), free prostate specific antigen (fPSA), and the fPSA/tPSA ratio in individuals with PCa aligns with corresponding clinical manifestations. There was no statistically significant disparity observed in the levels of high-density lipoprotein (HDL), low-density lipoprotein (LDL), triglycerides (TG), and total cholesterol (TC) between the group diagnosed with BPH and the group diagnosed with PCa.

**Table 1:** A comparative analysis of clinical baseline data for patient-related test indicators.

Characteristics	BPH (n=27)	Pca (n=27)	statistics	*p*
**Age(years)**	68.04 ± 11.74	71.00 ± 8.39	-1.05	0.30
**tPSA(ng/ml)**	3.22(1.18,9.78)	12.27(6.40,24.00)	-3.78	<0.001
**fPSA (ng/ml)**	0.77(0.25,1.43)	1.81(0.94,3.57)	-3.07	0.002
**f/tPSA (%)**	25.12 ± 12.65	14.97 ± 6.74	3.61	<0.001
**NEUT (10^9/L)**	3.68(2.90,4.53)	3.84(2.97,5.70)	-0.74	0.46
**LYMPH (10^9/L)**	1.79 ± 0.67	1.86 ± 0.70	-0.36	0.72
**NLR (%)**	1.99(1.52,3.15)	2.03(1.25,2.71)	0.06	0.96
**PLT (10^9/L)**	172.00(150.00,226.00)	191.00(170.00,223.00)	-1.07	0.29
**PLR (%)**	101.88(79.10,130.50)	109.33(82.68,137.84)	-0.49	0.63
**ALB (g/L)**	38.20(35.30,39.80)	38.10(35.20,41.70)	-0.24	0.82
**GLOB (g/L)**	25.57 ± 2.74	27.98 ± 4.22	-2.44	0.02
**A/G (%)**	1.48 ± 0.20	1.40 ± 0.31	1.16	0.25
**HDL (mmol/L)**	1.06 ± 0.25	1.15 ± 0.35	-1.01	0.32
**LDL (mmol/L)**	2.78(1.98,3.14)	2.47(2.02,3.01)	0.04	0.97
**TG (mmol/L)**	0.99(0.75,1.56)	1.01(0.87,1.38)	-0.72	0.48
**TC (mmol/L)**	3.00(3.56,5.06)	4.40(3.55,4.84)	-0.43	0.67
Biopsy Gleason score, n (%)				
**≤6**		7(25.93%)		
**3 + 4**		6(22.22%)		
**4 + 3**		4(14.81%)		
**4 + 4,3 + 5, 5 + 3**		7(25.93%)		
**9 to 10**		3(11.11%)		

tPSA, Total prostate specific antigen; fPSA, Free prostate specific antigen; f/tPSA, Free prostate specific antigen-to-Total prostate specific antigen ratio; NEUT, neutrophil; LYMPH, lymphocyte; NLR, Neutrophil-to-Lymphocyte Ratio; PLT, Platelet; PLR, Platelet -to-Lymphocyte Ratio; ALB, Albumin; GLOB, Globulin; A/G, Albumin-to-Globulin; HDL, High-density lipoprotein; LDL, Low-density lipoprotein; TG, Triacylglycerol; TC, Total Cholesterol.

### Urine screening results for differential proteins

**Figure 1:**
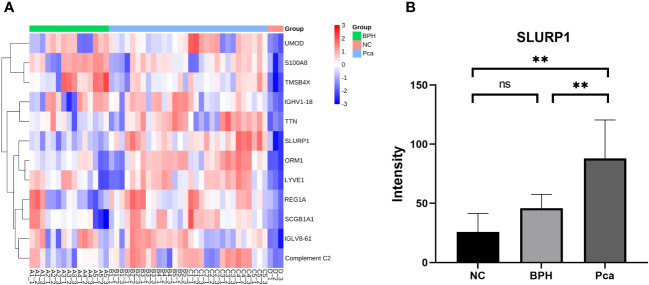
Differential protein screening in PCa, BPH, and normal human urine. **(A)** PCa screening for differentially expressed proteins. **(B)** An analysis of the expression of SLURP1 in PCa, BPH, and healthy controls. PCa group: Pca; BPH group: BPH; Normal control: NC, ns, *P*>0.05, **,*P*<0.01.

The utilization of the Uniprot database and MaxQuant software for the analysis of mass spectrometry data yielded findings indicating the identification of 7840 peptide segments and 1065 proteins. A comparative examination was conducted among the PCa group, BPH group, and normal group. **Figure 1A** illustrates the presence of 12 upregulated proteins that exhibited differential expression in the PCa group. Moreover, we conducted an analysis on the expression of SLURP1 across various cohorts, as illustrated in **Figure 1B**. The SLURP1 protein exhibited a progressive elevation in the urine samples obtained from individuals with normal prostate function, BPH, and PCa. Notably, a statistically significant distinction (*P*<0.05) was observed when comparing the SLURP1 protein levels in urine between patients diagnosed with BPH and those with PCa.

### Urine SLURP1 differential protein validation results

**Figure 2:**
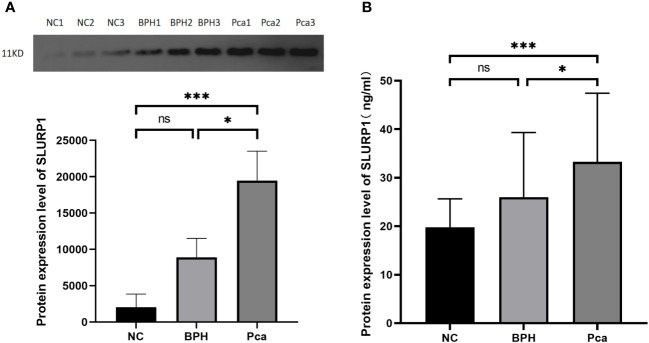
SLURP1 protein expression in urine samples from PCa, BPH, and normal controls. **(A)** Western blot strip quantitative analysis (grayscale value calculation). PCa group: Pca; BPH group: BPH; Normal control: ns, *P*>0.05, *,*P*<0.05, ***,*P*<0.001. **(B)** ELISA measures the level of SLURP1 protein in urine in ng/mL using a quantitative method. PCa group: Pca; BPH group: BPH; Normal control: NC. In each group, there are 27 samples. ns, *P*>0.05, *,*P*<0.05, ***,*P*<0.001.

Western blot analysis was conducted on the SLURP1 protein to ascertain its expression in urine across all groups. Subsequently, statistical analysis was performed on the grayscale values of the bands, as depicted in **Figure 2A**. ELISA detection and validation of the SLURP1 protein in urine were carried out, encompassing the PCa group (Pca=27), BPH group (BPH=27), and normal control group (NC=27), as illustrated in **Figure 2B**. In the Western blot analysis and ELISA detection validation results, the expression of SLURP1 in the urine of patients with PCa group was found to be significantly increased compared to both the normal control group and the BPH group, with statistical significance (*P*<0.05). Furthermore, the observed expression of SLURP1 aligns with the findings from mass spectrometry preliminary screening, suggesting that the SLURP1 protein in urine exhibits favorable specificity and reproducibility as a target protein for the differential diagnosis of PCa.

### SLURP1 expression levels in tissue samples

**Figure 3:**
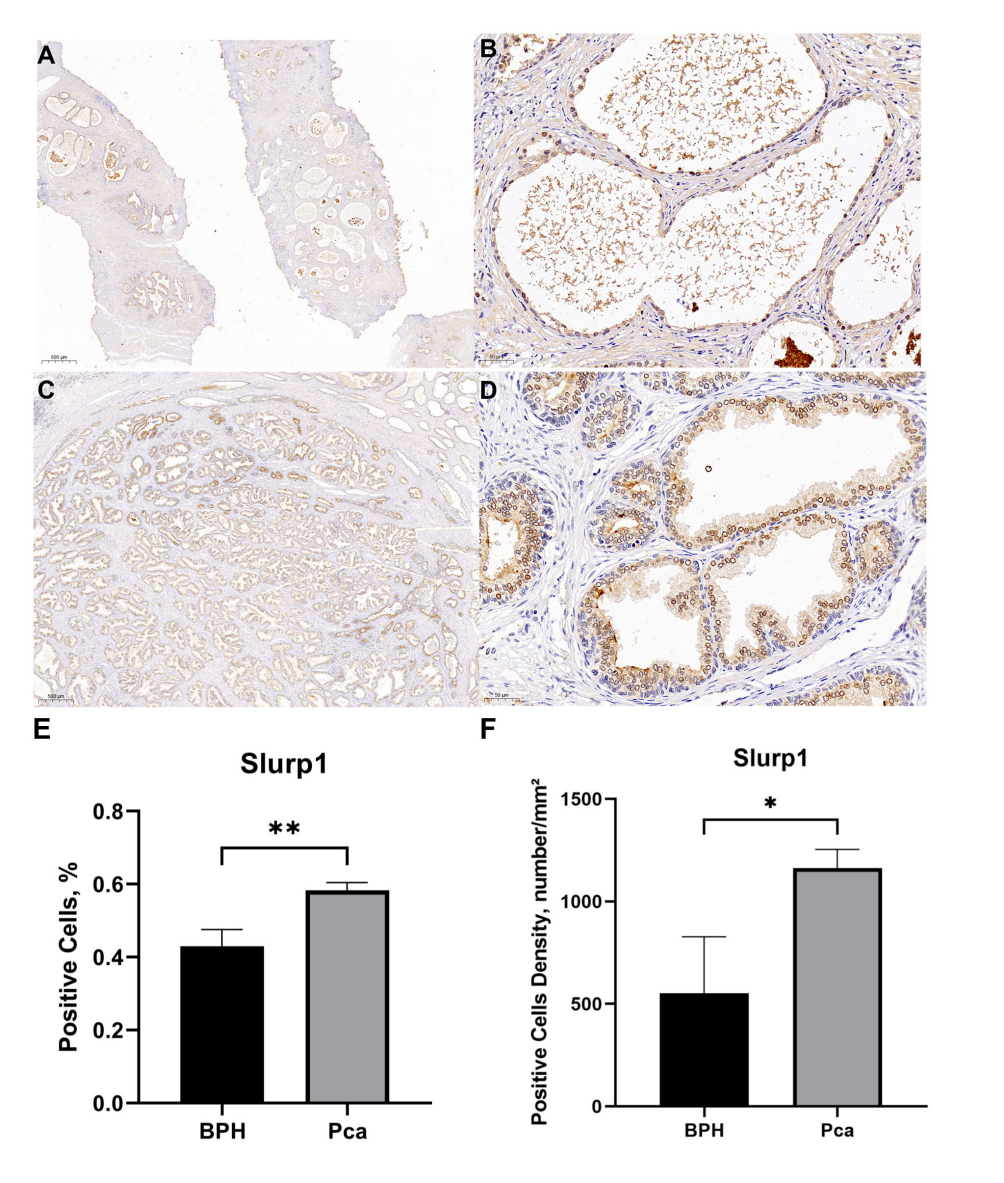
Comparison of SLURP1 expression levels in PCa and BPH tissue samples. **(A)** The expression of SLURP1 protein in BPH tissue(Bar=500μm). **(B)** The expression of SLURP1 protein in BPH tissue (Bar=50μm). **(C)** The expression of SLURP1 protein in PCa tissue (Bar=500μm). **(D)** The expression of SLURP1 protein in PCa tissue(Bar=50μm). **(E)** An analysis of the ratio of SLURP1- positive cells in PCa and BPH tissues, PCa group: Pca; BPH group: BPH. In each group, there are 3 samples. **,*P*<0.01. **(F)** An analysis of positive cell density in PCa and BPH tissues, PCa group: Pca; BPH group: BPH. In each group, there are 3 samples. *,*P*<0.05.

The positive cell rates in the pathological tissue samples of three cases of PCa were 56.17%, 58.50%, and 59.51%, respectively. The positive cell densities were 1150mm², 1260mm², and 1076mm². Similarly, the positive cell rates in the pathological tissue samples of three cases of BPH were 44.16%, 46.99%, and 37.69%, respectively. The positive cell densities were 279mm², 827mm², and 554mm².According to the findings presented in **Figure 3**, there was a notable increase in the positive expression rate of the SLURP1 protein in PCa tissue when compared to BPH tissue. This disparity was found to be statistically significant (*P*<0.05).

### An analysis of SLURP1 and PSA correlations

**Figure 4:**
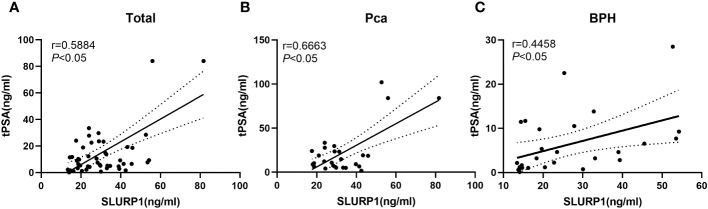
An analysis of the correlation between urine SLURP1 levels and clinical indicators of PCa and BPH. **(A)** Correlation analysis between the overall urine concentration of SLURP1 and clinical indicators of blood PSA in patients with BPH and PCa. **(B)** Correlation analysis between the concentration of SLURP1 in urine and clinical indicators of blood PSA in patients with PCa. **(C)** Correlation analysis between the concentration of SLURP1 in urine and clinical indicators of blood PSA in patients with BPH.

Using validation group data, we investigated the association between the urinary concentration of SLURP1 and clinical indicators such as blood PSA levels. **Figure 4** demonstrates a linear correlation between the urinary concentration of SLURP1 and blood PSA levels in both the PCa and BPH groups. Notably, an increase in the urinary concentration of SLURP1 may correspond to an elevation in PSA concentration.

### PCa diagnosis assisted by SLURP1 protein

**Figure 5:**
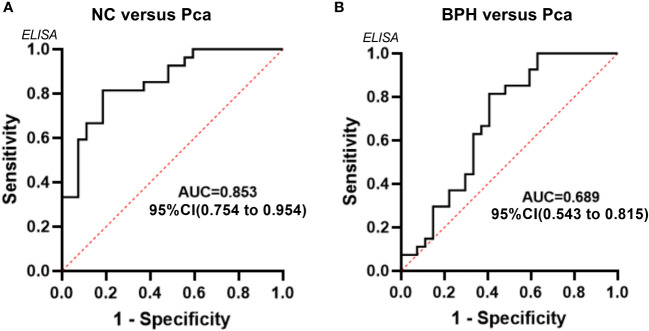
An analysis of the ROC curve for the SLURP1 protein in urine samples from PCa, BPH, and normal controls. **(A)** ROC curves using ELISA data obtained from the normal control group and PCa group within the validation queue. **(B)** ROC curves using ELISA data derived from the BPH group and PCa group within the validation queue.

Based on the data from the validation group, an ROC curve was established to assess the diagnostic effectiveness of SLURP1 in detecting PCa in urine. **Figure 5** illustrates that the AUC for diagnostic efficacy in distinguishing normal individuals from the PCa group is 0.853, with a 95% confidence interval ranging from 0.754 to 0.954. Additionally, the Youden index is reported as 0.810, indicating a sensitivity and specificity of 0.815. Furthermore, the AUC for diagnostic efficacy in differentiating between BPH and PCa is 0.689, with a 95% confidence interval of 0.543 to 0.815. The corresponding Youden index is 0.407, with a sensitivity of 0.815 and specificity of 0.593. In conclusion, SLURP1 demonstrates auxiliary potential in PCa detection.

## Discussion

Pca, a malignancy originating from epithelial cells in the prostate gland, ranks among the most prevalent tumors within the male urogenital system. Characterized by a gradual progression, this disease often remains asymptomatic during its initial stages (19). Consequently, early detection through screening holds paramount importance for both individuals with a familial predisposition to PCa and the general populace. However, the current rate of PCa screening coverage remains unsatisfactory (20). Urine proteomics analysis offers a convenient, non-invasive, safe, and reliable approach, characterized by high patient compliance, thereby enhancing the screening coverage for PCa (21).

This study utilized urine proteomics technology to construct a distinct expression profile of urine proteins in individuals without any medical conditions, individuals with BPH, and individuals with PCa. The SLURP1 protein exhibited a progressive upward trajectory in the urine of individuals without any medical conditions, individuals with BPH, and individuals with PCa, with statistically significant disparities. Additional validation was performed utilizing Western blot and ELISA techniques. In comparison to individuals with BPH and those without any prostate abnormalities, the urinary expression of SLURP1 protein was found to be significantly elevated in patients diagnosed with PCa. A statistically significant disparity was observed, aligning with the outcomes of the mass spectrometry screening. Further application of immunohistochemistry methodology revealed a notable increase in the expression level of the SLURP1 protein in PCa tissue, when compared to BPH. This finding is consistent with the observed expression pattern in urine. In the present investigation, we have additionally observed a positive association between the urinary expression level of SLURP1 and the serum prostate specific antigen (PSA). The urinary SLURP1 protein may serve as a biomarker for PCa screening, with clinical application potential.

The SLURP1 protein, a member of the Ly-6 family, does not possess GPI anchoring signals. It is secreted by basal keratinocytes and can be found in the bloodstream and urine. Remarkably, SLURP1 represents the inaugural member of the secreted Ly-6 protein family to be observed in mammals (10). SLURP1 exerts its influence on immune cells via autocrine and paracrine signaling pathways. Additionally, SLURP1 is involved in various cellular processes, including cell membrane adhesion, growth, migration, differentiation, and tumorigenesis of epithelial cells (22).The differential expression of SLURP1 has been observed in various diseases. In esophageal squamous cell carcinoma, colorectal cancer, melanoma, and lung cancer, the expression of SLURP1 is notably downregulated and is associated with disease staging (23–26). Conversely, in psoriasis lesions, the expression of SLURP1 is elevated compared to that in healthy individuals, potentially indicating its role in regulating the proliferation and differentiation of keratinocytes (27, 28). This study has identified elevated concentrations of the SLURP1 protein in both urine and tissues of individuals diagnosed with PCa, potentially indicating a correlation between SLURP1 and the progression and spread of PCa.

The majority of patients diagnosed with advanced and recurrent PCa demonstrate allelic acquisition and amplification of genes located in the chromosome 8q region, such as the oncogene c-myc situated at the proximal end of 8q24 (29). Both SLURP1 and prostate stem cell antigen (PSCA) are members of the LY6 gene protein family and are situated within the 8q24 chromosome region (30). The expression of PSCA is predominantly observed in prostate tissue, with a notably high expression in PCa tissue and peripheral blood (31), aligning with our research findings on SLURP1.The occurrence and malignant progression of PCa are closely associated with the process of epithelial-mesenchymal transition. This transition leads to the loss of polarity in epithelial cells and disruption of intercellular connections within the epithelium. Consequently, the transformed epithelial cells acquire tumor-like characteristics, including metastatic and migratory properties (32).

According to research, PSCA promotes cell proliferation by upregulating the genes c-myc, cyclin D1, and cyclin E2 in PCa cells via the PI3K/AKT pathway (33). The activation of p38/NF-κB signaling pathway by PSCA induces the release of IL-6, thereby facilitating epithelial-mesenchymal transition in PCa and influencing its invasive and metastatic properties (34). There is a significant correlation between PSCA expression in PCa tissue and unfavorable prognostic factors such as Gleason grading and extraprostatic invasion (35). According to recent research reports, there is a notable increase in the levels of PSCA protein and mRNA expression in poorly differentiated PCa tissues compared to moderately differentiated and well differentiated PCa tissues. This finding indicates a positive association between the expression level of PSCA and the tumor grade (36).

The observed upregulation of SLURP1 in PCa aligns with the expression pattern of PSCA. Consequently, we hypothesize that the heightened levels of SLURP1 within the 8q24 chromosome region potentially play a role in modulating the process of epithelial mesenchymal transition. This transition facilitates the acquisition of mesenchymal traits by epithelial cells, thereby enabling PCa cells to detach from the primary lesion and disseminate through the bloodstream or lymphatic system, ultimately culminating in tumor invasion and metastasis. Additionally, we found a positive correlation between SLURP1 expression and PSA serum levels in this study. With increasing malignancy, the tumor’s invasiveness increases, and the more PSA enters the blood, the higher its concentration (37). This discovery provides additional evidence supporting our hypothesis that there exists a significant association between SLURP1 and the progression of PCa, establishing a solid groundwork for the identification of precise biomarkers for PCa diagnosis.

This study was constrained by the limited number of enrolled patients. As a result, the pathological tissue samples are inadequate too. In the future research, we will enroll large scale patients from multiple medical centers. Collect more samples and validate the marker in various levels. We will explore the relationship between various stages of prostate cancer pathology and the SLURP1 protein expression. It is crucial to assess the reliability and effectiveness of the biomarkers identified in this study before their clinical use. Consequently, this research holds potential for future utilization in the realm of early detection of PCa through screening procedures.

## Conclusions

In summary, the clinical utility of the urine SLURP1 protein in the context of PCa screening is noteworthy. In contrast to the blood-based PSA test, the identification of SLURP1 in urine via non-invasive and cost-effective methods offers distinct advantages in facilitating early detection of PCa. Consequently, SLURP1 emerges as a promising urinary biomarker for PCa.

## Data availability statement

The raw data supporting the conclusions of this article will be made available by the authors, without undue reservation.

## Ethics statement

The present study adhered to the principles outlined in the Declaration of Helsinki. Approval for conducting the research was obtained from the Ethics Committee of Beijing Shijitan Hospital of Clinical Medicine(sjtkyll-lx-2021(115)). Furthermore, informed consent was duly obtained from all individual participants who were included in the study.

## Author contributions

TY: Conceptualization, Data curation, Formal analysis, Investigation, Methodology, Validation, Writing – original draft. TLiu: Data curation, Investigation, Formal analysis, Writing – review & editing. TLei: Data curation, Formal analysis, Writing – review & editing. TLi: Investigation, Writing – review & editing. NL: Resources, Writing – review & editing. MZ: Conceptualization, Methodology, Project administration, Funding acquisition, Resources, Supervision, Writing – review & editing.
